# Book Reviews

**Published:** 1872-09-15

**Authors:** 


### Book Reviews.

Clinical Lectures on the Diseases of Women, by Sir James Y. Simpson, Bart., M. D., etc. Edited by Alexander R. Simpson, M. D. D. Appleton & Co., New York.

This is a volume of 780 pages, made up of a series of Clinical Lectures, most of which were published in the *Medical Times and Gazette*, during the years 1859–1861. Ten out of the fifty lectures, however, drawn up from the editor's own notes and those of Dr. Black, with the aid of the author's lecture notes, are now published for the first time. The lectures being clinical are not arranged in any systematic order, but a copious table of contents, and a full index are introduced to facilitate reference. The following is a list of topics treated in the various lectures: Diagnosis of the Diseases of Women—Vesico Vaginal Fistula—Pelvic Cellulitis—Pelvic Peritonitis—Peri-Uterine or Pelvic Hematocele or Hematoma—Cancer of the Uterus—Carcinoma of the Uterus and Mamma—Coccygodynia and Diseases and Deformities of the Coccyx—Dysmenorrhœa—Closures and Contractions of the Vagina—Caruncles of the Urethra—Neuromata of the Vulva—Hyperæsthesia and Neuralgia of the Vulva; Abscess of the Labia Pudendi, and the Various Forms of Vulvitis—Surgical Fever; Its Etiology and Semeiology; Its Treatment—Phlegmasia Dolens—Spurious Pregnancy or Pseudocyesis—Its Prognosis, Pathology, and Treatment—Ovarian Dropsy; Symptoms, Diagnosis, and Treatment—Its Surgical Treatment—Injection of Iodine—Ovariotomy—Cranioclasm—Dropsy and Other Diseases of the Fallopian Tubes—Puerperal Mania—Puerperal Hypochondriasis, Sub-Involution of the Uterus after Delivery—Amenorrhœa—Rupture of Perineum—Fibroid Tumors of the Uterus; Their Differential Diagnosis, Prognosis and Treatment—Polypi of the Uterus—Leucorrhœa—Chronic Metritis—Prolapsus Uteri—Retroversion of Uterus. The work is illustrated by about 150 cuts, representing the various operations and morbid conditions described in the text.

Transactions of the Medical Society of the State of Pennsylvania at its Twenty-Third Annual Session, held at Franklyn, Pa., June, 1872, Vol. IX, Part 1.

This is a volume of 250 pages, and contains a large number of valuable reports and essays.

Proceedings of the American Association for the Advancement of Science—Twentieth Meeting—Held at Indianapolis, Indiana, August, 1871. Published by Joseph Lovering, Cambridge, 1872.

A volume containing many reports and contributions of interest and importance to the scientist.

New Treatment of Venereal Diseases and of Ulcerative Syphilitic Affections by Iodoform. Translated from the French of Dr. A. A. Izard, by Howard F. Damon, M. D. Boston: James Campbell, Publisher. Price fifty cents.

A pamphlet of 72 pages. A number of clinical observations are given, illustrative of the value of the new form of treatment.

